# Editorial: Decreasing the Impact of Treatment Resistance in Schizophrenia: Identifying Novel Molecular Targets/Pathways to Increase Treatment Efficacy

**DOI:** 10.3389/fphar.2020.01001

**Published:** 2020-07-02

**Authors:** Mirko Manchia, Bernardo Carpiniello

**Affiliations:** ^1^ Section of Psychiatry, Department of Medical Sciences and Public Health, University of Cagliari, Cagliari, Italy; ^2^ Unit of Clinical Psychiatry, University Hospital Agency of Cagliari, Cagliari, Italy; ^3^ Department of Pharmacology, Dalhousie University, Halifax, NS, Canada

**Keywords:** omics, epigenetics, pharmacogenetics, CYP, risk prediction, microbiota

Schizophrenia (SCZ) is a severe mental disorder with a prevalence of about 1% in the general population (Owen et al., 2016). Antipsychotics remain the mainstay for the treatment of core symptoms of SCZ. However, a substantial proportion of patients (20–30%) present little or no response to these treatments, with naturalistic data pointing to even higher rates of treatment resistant schizophrenia (TRS) (>50%) in community mental health settings (Beck et al., 2019). In addition, even an higher rate of patients shows suboptimal response to antipsychotics (Samara et al., 2019). This population of patients is heavily burdened by higher rates of persistent symptoms, longer duration of hospital admissions and higher treatment costs compared to patients responsive to antipsychotics (Carbon and Correll, 2014). TRS patients show more severe psychopathology, more impaired cognitive functioning, and poorer psychosocial adjustment, which result in worst community functioning, compared to non-TRS patients (Iasevoli et al., 2016). This makes the clinical and socioeconomic impact of TRS enormous. In this context, the early identification of individuals who might present treatment resistance in any phase of the illness course is vital. However, predictive models, either based on clinical or biological data, have not reached yet the accuracy threshold needed to be tested (and eventually validated) in clinical settings. It is conceivable that only models informed by multiple sources of data (phenotypic, genomic, transcriptomic, epigenomic, microbiome) will reach clinically relevance. For instance, a recent study showed that predictive methods, machine learning and polygenic risk scores (PRS), applied to genomic data were unable to discriminate with sufficient accuracy TRS patients form healthy controls (Vivian-Griffiths et al., 2019).

In this scenario, we believed to be timely a Research Topic summarizing the state of the art of “omics”, neurobiological and clinical research in the area of TRS. A series of studies focused on the role of genetic determinants in modulating TRS (Sagud et al.; Pisanu and Squassina; Vita et al.), response to antipsychotics, including to the mainstay treatment for TRS, clozapine (Bosia et al.; Koopmans et al.; Numata et al.), as well as the onset of side effects, such as clozapine-induced agranulocytosis and tardive dyskinesia (Numata et al.; Zai et al.). Specifically, Sagud et al. investigated whether haplotypic and genotypic association of COMT rs4680 and rs4818 polymorphisms modulated the risk of TRS in 931 Caucasian patients diagnosed with SCZ, of whom 270 met the criteria for TRS. They found a gender specific effect of COMT rs4680 and rs4818 polymorphisms with women carrying the G-allele having a lower risk of TRS (Sagud et al.). Pisanu and Squassina performed a critical appraisal of the literature on genetics of TRS, focusing on the ability of genomic data to inform predictive models of TRS. These authors found a lack of genetic markers showing adequate prediction accuracy in TRS. Furthermore, they highlighted the presence of contrasting findings on the power of PRS and machine-learning approaches in discriminating between TRS and non-TRS individuals (Pisanu and Squassina). Vita et al. performed a systematic review with the aim of summarizing the genetic and neuroimaging correlates of TRS and uncovering the underlying neurobiological mechanisms of such resistance. The authors showed that, although interesting data have come from (pharmaco)genetics and neuroimaging investigations as well as from the analysis of their interacting effects, few converging findings are available that describe the antipsychotic treatment response and resistance mechanisms in SCZ (Vita et al.).

Another set of studies focused on the role of genetic variation in predicting response to antipsychotics (Bosia et al.; Koopmans et al.; Numata et al.). Bosia et al. investigated the relationship between metabolic syndrome and cognition, testing for the moderating effect of the Sterol Regulatory Element Binding Transcription Factor 1 (SREBF-1) rs11868035 genetic polymorphism. To this end, they studied 172 outpatients with SCZ assessed for metabolic parameters and neurocognitive measures, with 138 patients, who completed cognitive remediation therapy (CRT), re-evaluated for cognition. They showed the negative impact of metabolic syndrome on executive functions and global cognitive outcome after CRT and revealed the significant effect of SREBF-1 polymorphism, with a higher prevalence of metabolic syndrome and worse processing speed performance among G/G homozygous subjects, compared to the A allele carriers (Bosia et al.). Koopmans et al. evaluated the effect of dose adjustment of antipsychotics to the CYP2D6 genotype and phenotype, in patients diagnosed with SCZ already receiving psychopharmacological treatment. These authors did not identify benefit for the patients from dose adjustment based on the CYP2D6 genotype or phenotype in terms both of efficacy and of safety (Koopmans et al.). Finally, Numata et al. examined the existing literature on genetic predictors of clozapine response. They highlighted that meta-analytical evidence indicate that only three SNPs (rs6313 and rs6314 in the HTR2A gene and rs1062613 in the HT3A gene) are significantly associated with clozapine response (Numata et al.). Concerning genetic predictors of side effects, Numata et al. also summarized genomic findings pointing to the association of genetic variants within the MHC region as well as to a series of other chromosomal regions, with the onset of clozapine induced agranulocytosis. Furthermore, Zai et al. showed a strong significant association of the G allele of the rs2445142 polymorphism within the Perlecan (HSPG2) gene with the risk of tardive dyskinesia.

The area of “omics” research was nicely complemented by the systematic review of Cuomo et al. investigating the possible relationships between microbiome, schizophrenia and treatment resistance. They also summarized the findings exploring the hypothesis that microbiome modulation with probiotics and prebiotics could influence illness severity and clinical response to antipsychotics (Cuomo et al.). This study confirmed that the microbiome is substantially altered in SCZ patients, with pathological reduced variability of the amount and distribution of its components (Cuomo et al.). Furthermore, it highlighted the lack of data on microbiome alteration in TRS as well as the presence of encouraging findings on the use of prebiotics/probiotics in SCZ (Cuomo et al.).

Neurobiological underpinnings of TRS were examined in three studies (Crocker and Tibbo; Rampino et al.; de Bartolomeis et al.). Crocker and Tibbo reviewed the evidence on the role of white matter abnormalities in the brain in the symptomology of psychotic disorders. They confirmed that white matter deficits correlate with treatment resistance in SCZ and proposed that putative myelin-enhancing therapies, including n-3 PUFA, minocycline, clemastine, and sulfasalazine among the others, would be potential candidates for large-scale clinical trials in SCZ as well as for targeting TRS (Crocker and Tibbo). Furthermore, Rampino et al. analyzed the analogies between the established dopaminergic pathophysiological hypothesis of SCZ and the available findings coming from candidate gene and genome-wide association studies. These authors observed that the prevalence of genes involved in dopamine transmission among those associated with treatment responsiveness in SCZ is flagrant and suggested that the further examination of genes that are more distal to dopamine signaling, and are nonetheless associated with SCZ risk and drug responsiveness, may provide a new roster of targets for drug development (Rampino et al.). Finally, de Bartolomeis et al. explored the role of glycine, a non-essential amino acid that plays a critical role in both inhibitory and excitatory neurotransmission, and its related pathways in TRS. These authors highlighted that converging findings point to the pharmacological augmentation of glutamatergic transmission through glycine signaling enhancement as a valuable option to restore the function of prefrontal cortex to control dopamine release, offering a potentially useful strategy in TRS (de Bartolomeis et al.).

Finally, a set of studies focused on the role of clinical determinants of TRS (Thomas-Brown et al.; Barlati et al.; Bozzatello et al.). Thomas-Brown et al. reported on the loss of efficacy of antipsychotic drugs on sleep when concomitant use of cannabis was present, showing that in these cases risperidone was more beneficial than haloperidol in improving sleep efficiency. Barlati et al. systematically reviewed the evidence on factors that can modulate the response to cognitive remediation (CR) interventions in SCZ. The authors identified a series of predictors, such as age, duration of illness, premorbid adjustment, baseline cognitive performance, intrinsic motivation, hostility among the others, that can inform personalized approaches to cognitive remediation in SCZ and can increase the rates of treatment responsiveness (Barlati et al.). Finally, Bozzatello et al. systematically reviewed the evidence on clinical predictors of TRS. They found that a series of factors such as lower premorbid functioning, lower level of education, negative symptoms from first psychotic episode, comorbid substance use, younger age at onset, lack of early response, non-adherence to treatment, longer duration of untreated psychosis, can inform treatment decision making and lead to a personalized approach in TRS (Bozzatello et al.).

In conclusion, this Research Topic has offered a perspective of the most promising advancements in the area of TRS. These data might guide the development of predictive tools of TRS, and help identifying new molecular targets and pathways to expand the pharmacological armamentarium. This is crucial in psychiatry, where the pharmacological pipeline has lagged behind in the past decade, and it is even more important in the presence of treatment resistance, particularly in a severe and chronic mental illness such as SCZ.

## Author Contributions

MM and BC took part in drafting the article, revising it critically for important intellectual content, gave final approval of the version to be published, and agreed to be accountable for all aspects of the work.

## Funding 

This paper was partly funded by Fondo Integrativo per la Ricerca (FIR)-2019 granted to MM and BC.

## Conflict of Interest

The authors declare that the research was conducted in the absence of any commercial or financial relationships that could be construed as a potential conflict of interest.

## References

[B1] Beck K. McCutcheon R. Stephenson L. Schilderman M. Patel N. Ramsay R. (2019). Prevalence of treatment-resistant psychoses in the community: A naturalistic study. J. Psychopharmacol. 33, 1248–1253. 10.1177/0269881119855995 31241396

[B2] Bozzatello P. Bellino S. Rocca P. (2019). Predictive factors of treatment resistance in first episode of psychosis: A systematic review. Front. Psychiatry 10, 1–14. 10.3389/fpsyt.2019.00067 30863323 PMC6399388

[B3] Carbon M. Correll C. U. (2014). Clinical predictors of therapeutic response to antipsychotics in schizophrenia. Dialogues Clin. Neurosci. 16, 505–524. 25733955 10.31887/DCNS.2014.16.4/mcarbon PMC4336920

[B4] Iasevoli F. Giordano S. Balletta R. Latte G. Formato M. V. Prinzivalli E. (2016). Treatment resistant schizophrenia is associated with the worst community functioning among severely-ill highly-disabling psychiatric conditions and is the most relevant predictor of poorer achievements in functional milestones. Prog. Neuropsychopharmacol. Biol. Psychiatry 65, 34–48. 10.1016/j.pnpbp.2015.08.010 26320028

[B5] Owen M. J. Sawa A. Mortensen P. B. (2016). Schizophrenia. Lancet 388, 86–97. 10.1016/S0140-6736(15)01121-6 26777917 PMC4940219

[B6] Samara M. T. Nikolakopoulou A. Salanti G. Leucht S. (2019). How many patients with schizophrenia do not respond to antipsychotic drugs in the short term? An analysis based on individual patient data from randomized controlled trials. Schizophr. Bull. 45, 639–646. 10.1093/schbul/sby095 29982701 PMC6483567

[B7] Vivian-Griffiths T. Baker E. Schmidt K. M. Bracher-Smith M. Walters J. Artemiou A. (2019). Predictive modeling of schizophrenia from genomic data: Comparison of polygenic risk score with kernel support vector machines approach. Am. J. Med. Genet. B. Neuropsychiatr. Genet. 180, 80–85. 10.1002/ajmg.b.32705 30516002 PMC6492016

